# ARNS: Adaptive Relay-Node Selection Method for Message Broadcasting in the Internet of Vehicles

**DOI:** 10.3390/s20051338

**Published:** 2020-02-29

**Authors:** Dun Cao, Yuchen Jiang, Jin Wang, Baofeng Ji, Osama Alfarraj, Amr Tolba, Xiaomin Ma, Yonghe Liu

**Affiliations:** 1 School of Computer and Communication Engineering, Changsha University of Science and Technology, Changsha 410114, China; caodun@csust.edu.cn (D.C.); a13789010512@gmail.com (Y.J.); 2 College of Information Engineering, Henan University of Science and Technology, Luoyang 471000, China; baofengji@haust.edu.cn; 3 Computer Science Department, Community College, King Saud University, Riyadh 11437, Saudi Arabia; oalfarraj@ksu.edu.sa (O.A.); atolba@ksu.edu.sa (A.T.); 4 Mathematics and Computer Science Department, Faculty of Science, Menoufia University, Shebin-El-kom 32511, Egypt; 5 College of Science and Engineering, Oral Roberts University, Tulsa, OK 74171, USA; xma@oru.edu; 6 Department of Computer Science and Engineering, University of Texas at Arlington, Arlington, TX 76019, USA; yonghe@cse.uta.edu

**Keywords:** Internet of Vehicles, adaptive mechanism, multi-hop broadcasting, relay-node selection

## Abstract

The proper utilization of road information can improve the performance of relay-node selection methods. However, the existing schemes are only applicable to a specific road structure, and this limits their application in real-world scenarios where mostly more than one road structure exists in the Region of Interest (RoI), even in the communication range of a sender. In this paper, we propose an adaptive relay-node selection (ARNS) method based on the exponential partition to implement message broadcasting in complex scenarios. First, we improved a relay-node selection method in the curved road scenarios through the re-definition of the optimal position considering the distribution of the obstacles. Then, we proposed a criterion of classifying road structures based on their broadcast characteristics. Finally, ARNS is designed to adaptively apply the appropriate relay-node selection method based on the exponential partition in realistic scenarios. Simulation results on a real-world map show that the end-to-end broadcast delay of ARNS is reduced by at least 13.8% compared to the beacon-based relay-node selection method, and at least 14.0% compared to the trinary partitioned black-burst-based broadcast protocol (3P3B)-based relay-node selection method. The broadcast coverage is increased by 3.6–7% in curved road scenarios, with obstacles benefitting from the consideration of the distribution of obstacles. Moreover, ARNS achieves a higher and more stable packet delivery ratio (PDR) than existing methods profiting from the adaptive selection mechanism.

## 1. Introduction

The Internet of Vehicles (IoV) can play an important role in reducing traffic pressure and improving driving safety. Relay-node selection is the basis of IoV, and has attracted significant attention from researchers in recent years [1,2,3,4,5,6,7,8,9,10,11,12,13,14,15,16,17,18,19,20,21,22,23,24,25,26,27,28,29,30,31,32,33]. By appropriately selecting relay-nodes to forward messages, we can expand the coverage of messages with high time efficiency. Such methods aim to select a relay-node quickly and cover more range in one hop.

Based on the difference in obtaining information of neighbor nodes, relay-node selection methods can be classified into beacon-based relay-node selection methods (called beacon-based methods) and black-burst-based relay-node selection methods (called black-burst-based methods). Beacon-based methods obtain the information of neighbor nodes by constructing a routing table with periodic flood beacons, while black-burst-based methods obtain the location information of nodes within the communication range of the sender by broadcasting a black-burst in real-time. Because of the utilization of real-time information, black-burst-based methods exhibit better performance. What is more, the beacon-based methods are prone to consume more bandwidth resources due to the periodical beacons [34,35,36,37,38,39,40], while the black-burst-based methods have few such problems.

Currently, most modeling and analysis of the relay-node selection [16,17,18] do not consider the influence of the complex road structure, and the black-burst-based methods [21,22,23,24,25,26] are only applicable to one specific road structure. However, there is usually more than one road structure in real-world Region of Interest (RoI) that messages need to cover; even diverse road structures exist in the communication range of a sender.

Therefore, we propose an adaptive relay-node selection method (ARNS) that is based on exponential partition suitable for complex road structures in the real world. In this paper, our contributions are summarized as follows:

- According to the specific distribution of obstacles in the real world, the optimal position-selection is redefined, and a curved road relay-node selection method suitable for the actual situations is proposed.
- A criterion of classifying road structures is proposed to judge the road structure in complex scenarios.
- Based on the above work, an adaptive relay-node selection method is designed to suit two real-world situations: the differences of the road structures in the communication range of the different senders, and multiple road structures in the communication range of one sender.

The rest of the paper is organized as follows: Section 2 briefly introduces related work on relay-node selection methods. The problems of message broadcasting in RoI, which include complex road structures and the impact of obstacles, are analyzed in Section 3. An adaptive relay-node selection method based on the exponential partition is presented in Section 4. Section 5 demonstrates the performance of ARNS compared to other methods, and finally, we draw conclusions in Section 6.

## 2. Related Work

Several methods are proposed for relay-node selection in IoV, as discussed in the following.

Greedy perimeter stateless routing (GPSR) [19] obtains the location of neighbor nodes through periodic flood beacons, and selects the relay-node in each hop using the greedy algorithm. When the greedy algorithm fails, the relay-node is selected with the right-hand rule. The advantage of GPSR is that it can be applied in all road structures. However, the information update of neighbor nodes in GPSR is not real-time, and it limits the performance. What is more, GPSR mainly considers end-to-end message propagation and does not fully consider message broadcasting. In order to improve the performance of message broadcasting, a real-time adaptive dissemination system (RTAD) is proposed in [20], and it defines two metrics—informed vehicles and messages received—and selects the most suitable beacon-based method for different RoIs based on the simulation results of the two metrics. Its advantage is that the message broadcasting in urban scenarios is achieved with better overall performance. However, it still has the problem of lacking real-time information, which is the same as GPSR and is only suitable in urban scenarios.

Urban multi-hop broadcast protocol (UMB) [21] is a black-burst-based relay-node selection method, which solves the problem of lacking real-time information in the beacon-based method. It aims to maximize message progress by selecting the farthest vehicle as the relay-node. The sender broadcasts a Request-To-Broadcast (RTB) packet in its communication range. Upon the reception of RTB, nodes, i.e., vehicles, broadcast a channel jamming signal, i.e., black-burst, for a duration that is proportional to the node’s distance from the sender. Then, the farthest node transmits the longest black-burst, and performs forwarding. The disadvantage of UMB is that it has a relatively high communication delay since it spends the longest black-burst to select the farthest node to perform forwarding. Binary-partition-assisted broadcast protocol (BPAB) [22] is a binary partitioning broadcast method based on the black-burst, and solves the problem of UMB. It deploys a binary partitioning scheme and a novel contention mechanism. The binary partitioning scheme iteratively divides the range, which is the communication range in the first iteration and the selected segment in other iterations, into multiple segments. In addition, the farthest segment which contains nodes is selected by the aid of the black-bursts. Then, through a novel contention mechanism, a node is randomly selected as the relay-node in the farthest segment. Compared with the previous methods, BPAB achieves a lower and more stable delay, but it only works on the straight road or the junction. Trinary partitioned black-burst-based broadcast protocol (3P3B) [23] is a trinary partitioning broadcast method. Improving on BPAB, 3P3B uses a trinary partitioning method instead of the binary partitioning, and introduces mini-DIFS in the channel access period before the start of relay-node selection to reduce the channel access delay. With these improvements, it achieves a lower delay than BPAB, but it only considers the relay-node selection in straight road scenarios. Exponent-based partitioning broadcast protocol (EPBP) [24] is an exponential partitioning broadcast method. Improving on 3P3B, it divides the communication range of sender into $N_{\mathrm{part}}$ segments for $N_{\mathrm{iter}}$ iterations. The width of segment increases exponentially with the increase of its distance from the relay-node’s optimal position. Then, a non-empty segment closest to the optimal position is selected as the final segment. Finally, a node in the final segment is randomly selected as the relay-node through an exponential back-off method. The delay of the partitioning process is called partition delay, and the delay of the exponential back-off process is called contention delay. Due to the exponential partition, EPBP has a lower and more stable delay than 3P3B. However, EPBP still is suitable for the straight road scenarios. In order to solve the problem, a complete EPBP-based curved road relay-node selection method is proposed in [25]. It implements relay-node selection in curved road scenarios through three modes: the normal selection, the reverse selection, and the double-direction selection. When a vacant appears in the normal selection, it will enter the double-direction selection. At this time, the reverse selection and the normal selection are performed simultaneously, and the farthest point from the sender in a vacant as the end point in the reverse selection. Through the three modes, it achieves a high broadcast coverage. However, it has a disadvantage in that it does not consider the influences of obstacles. Thus, an EPBP-based junction relay-node selection method is proposed in [26]. Improving on EPBP, it implements relay-node selection in junction scenarios with obstacles through two phases: the junction phase and the branch phase. It selects the node close to the center of the junction as the relay-node in the junction phase, and selects the furthest node on each branch as the relay-node in the branch phase. Compared to BPAB, it achieves a lower delay. However, it does not consider the situation where the branches are not a straight road.

Though these black-burst-based methods [21,22,23,24,25,26], including our EPBP-based work [24,25,26], show better performance compared to the beacon-based methods [19,20], they are only suitable for a certain road structure, e.g., the methods in [24,27] are only for straight roads, that in [26] are only for junctions, and in [25] are only for curved roads. However, in the real world, varied road structures may exist in RoI and multiple road structures in the communication range of the sender. Moreover, the distribution of the obstacles can affect the relay-node selection. Therefore, in this paper, we have designed ARNS by fully considering the above situations to achieve better robustness. In the next section, we will describe the scenarios and state the problems.

## 3. Scenario Description and Problem Statement

In real-world IoV, the selection of relay-nodes needs to consider the high mobility of vehicles, the diversity of road structures, and the existence of obstacles in RoI to achieve higher coverage with lower delay. EPBP and its derived methods can well solve the problem of real-time caused by the high mobility of vehicles, but it fails to completely solve the problem of broadcasting in RoI with various road structures and obstacles.

**Figure 1:**
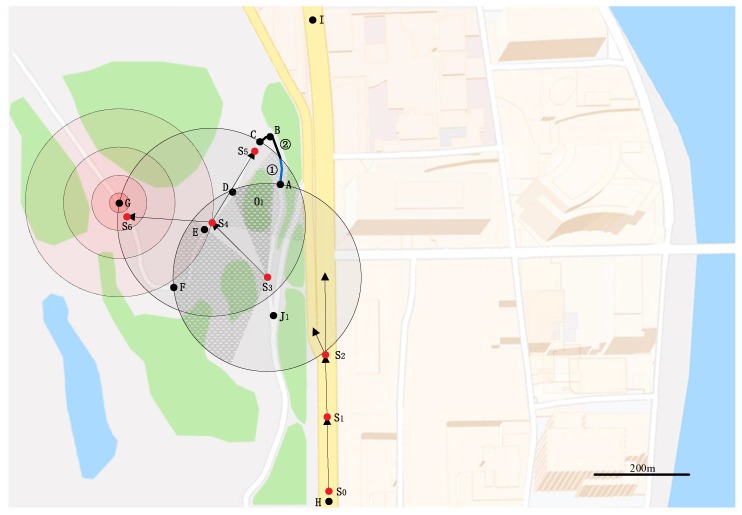
Relay-node selection in the Region of Interest (RoI).

For example, Figure 1 shows an area where various road structures and obstacles exist, and it is assumed to be an RoI of the message generated by Node S_0_ at Point H. The road structure on the west of the road section $\overset{⌢}{\mathrm{HI}}$ ($\overset{⌢}{\mathrm{HI}}$ indicates the road section connecting Point H and Point I is a curved road with a junction J_1_ and is surrounded randomly by green woods. Additionally, the road structures on the east of $\overset{⌢}{\mathrm{HI}}$ are the straight roads with junctions and there are buildings around these junctions. Woods and buildings are obstacles that can prevent the dissemination of messages. The message is expected to cover the RoI, so the ends of the road at the RoI boundary are the termination positions of broadcast.

A process of message broadcasting is illustrated in Figure 1. Node S_0_ is an original sender, and a message broadcasted by S_0_ is expected to cover the region shown in the map, i.e., the RoI of the message. Obviously, the road structures in the communication ranges of Node S_1_, S_2_, and S_4_ are the straight road, the junction, and the curved road, respectively, so the corresponding relay-node selection methods, i.e., the method in [24] for straight road scenarios, that in [26] for junction scenarios, and that for curved road scenarios in [25], are adopted according to the road structure. However, one problem needs to be solved, and that is how to distinguish road structures. Moreover, the road section in the communication range of one sender maybe consists of two or more road structures, not one typical road structure discussed in the existing works. This scenario is given as an example in Figure 1 as the road section in the communication range of Node S_3_. The range covers a junction and three curved road sections, neither the typical junction with several straight branches nor the typical curve only including the curved road section. Thus, in order to realize the node-selection in real-world scenarios, the first problem should be resolved as follows. 

Problem 1: how to classify the road structure?

The broadcasted message is expected to cover the whole RoI at the cost of as little time as possible. Thus, in one hop, the node at the farthest position from the sender in the direction of message broadcasting is the most favorite relay-node. The farthest position is defined as the optimal position [24]. In the real world, the obstacles will affect the location of the optimal position. The line-of-sight condition in straight road scenarios is good because no obstacle affects the communication range of the sender, thus existing relay-node selection methods [21,22,23,24] use the point farthest from the sender as the optimal position on the straight road scenarios. In junction scenarios, obstacles such as buildings generally exist near junctions, and the existing relay-node selection methods [21,22,26] applicable for junction scenarios select a node close to the center of the junction as the relay-node of the first hop, and achieve the maximum coverage of all branches with the second hop to complete message broadcasting. In curve scenarios, the general relay-node selection methods [13,14] consider that obstacles are generally around road corners, so the corner of the curved road is marked as the optimal position to eliminate the impact of obstacles on the message broadcasting. However, in the specific scenarios, the effect of obstacles on the location of the optimal position needs to be analyzed differently. As shown in Figure 1, the road section $\overset{⌢}{\mathrm{BF}}$ is out of the sight of Point A due to the blocking by Obstacle O_1_, so the sender at Point A can only use corner Point B as the optimal position to realize the relay-node selection in this curved road scenario. However, the road section $\overset{⌢}{\mathrm{EG}}$ has a good line-of-sight condition because of no blocks, so the sender at Point E can directly select the farthest Point G in its coverage area as the optimal position. Therefore, by considering the specific distribution of obstacles within the communication range, we can select the proper optimal position to achieve the maximum coverage of one-hop and reduce the delay of the relay-node selection. Thus, the second problem to be resolved is described as follows.

Problem 2: how to determine the optimal position?

As shown in Figure 1, there are two road sections that are not covered by the broadcast: one is road section ① indicated by the blue solid line, which is within the communication range of Node S_4_, but not covered by the signal of Node S_4_ because of the obstruction of Obstacle O_1_; another is road section ② indicated by the black solid line, which is outside the communication range of Node S_3_ and S_4_. As we aim to achieve full coverage of RoI, the location of the optimal position ensures that the broadcasting message can cover these road sections, i.e., road section ① and ②. 

It should be noted that we only consider relay-node selection in vehicle to vehicle (V2V), and nodes can obtain not only their own position by using GPS, but also the local information about roads and obstacles by using GIS.

To solve the problems of relay-node selection in the scenarios described above, in the next section, we propose an adaptive relay-node selection method that adaptively selects a relay-node selection method suitable for the current scenario according to the road structures and obstacles within the communication range of the sender.

## 4. Method Design

In this section, we will propose ARNS to solve the problems described in Section 3, but before that, we need to improve the EPBP-based methods to make them suitable for real-world scenarios. Therefore, the content of this section is organized as follows: we first propose an EPBP-based relay-node selection method suitable for curved road scenarios with obstacles, then, develop a criterion of classifying road structures. Moreover, we improve the EPBP-based junction relay-node selection method [26] to resolve the problems of multiple road structures existing in the communication range of the sender. Finally, an adaptive relay-node selection method based on these above works is proposed. The goal of this method is to achieve full coverage of RoI with the lowest delay.

### 4.1. EPBP-Based Relay-Node Selection Method Suitable for Curved Road Scenarios with Obstacles

Based on the analysis in Section 3, we first define Optimal Position and Vacant to facilitate the description of the relay-node selection method in curved road scenarios with obstacles.

**Definition** **1.** *Optimal Position $P_{\mathrm{opt}}\in \left\{Node_{1}\right\}\cup \left\{Node_{2}\right\}$ is the point that is closest to the terminal point of the curved road in the direction of message broadcasting, where $\left\{Node_{1}\right\}$ is the set of the intersections of the sender’s communication boundary and the curved roads that are not blocked by obstacles; $\left\{Node_{2}\right\}$ is the set of the intersections of the curved road and the tangents to the profile of the obstacles from the sender.*

**Definition** **2.** *Vacant is the segment of the curved road that is not covered by the communication ranges of the sender and the relay-node because of the high curving rate of the curved road and the blocking by the obstacles.*

Taking Figure 1 as an example, road section ① and ② are both vacant, because road section ① is not covered by the signal of Node S_4_ due to the obstruction of Obstacle O_1_, and road section ② is not within the communication range of Node S_3_ and S_4_.

Next, we improved the reverse selection [25] to solve the problem of vacant-caused reduction of broadcast coverage. When a sender finds that there is a vacant between itself and the sender in the previous hop, it enters the reverse selection. At this time, it serves as an initial sender of the reverse selection and broadcasts an RTB packet to start the normal selection and the reverse selection simultaneously. The reverse selection chooses the nearest corner to the initial sender in the reverse direction as the optimal position, and the endpoint of the vacant closest to the previous sender as the termination of the reverse selection. In the reverse direction, only the reverse selection continues until it completely covers the vacant. To distinguish three states of relay-node selection—only the normal selection, only the reverse selection, and the concurrence of the normal selection and the reverse selection, we added a mode flag into the RTB packet. Moreover, we assigned black-bursts with different frequencies to avoid interfering with each other between nodes in different states. Based on the above definitions and descriptions, we propose an EPBP-based relay-node selection method suitable for curved road scenarios with obstacles. The pseudo code is as follows in Algorithm 1.

**Algorithm 1.** Method of Relay-Node Selection on Curved Roads1**Input:** Set of Nodes N, Obstacles Ο.2**Input:** Message sender S, previous sender $S_{\mathrm{pre}}$, terminal point $P_{\mathrm{end}}$.3**Output:** Relay-node on curved roads.4**Phase 1. Judgment Phase:**5 **if** there is an area between S and $S_{\mathrm{pre}}$ that is blocked by Ο or out of the communication range of S and $S_{\mathrm{pre}}$.6  Determine the area as a vacant.7**Phase 2. RTB Packet Broadcast Phase:**8 **if** there is a vacant between S and $S_{\mathrm{pre}}$
9  Set the mode flag of RTB packet to 3 (means simultaneously start the normal selection and the reverse selection).10  Determine the optimal position $P_{\mathrm{opt}\_\mathrm{norm}}$ in the message propagation direction according to **Definition 1.**11  Choose the nearest corner as the optimal position $P_{\mathrm{opt}\_\mathrm{rev}}$ in the reverse direction.12  Determine the endpoint of the vacant closest to $S_{\mathrm{pre}}$ as the termination of the reverse selection $P_{\mathrm{rev}\_\mathrm{end}}$.13  Add $P_{\mathrm{opt}\_\mathrm{norm}}$, $P_{\mathrm{opt}\_\mathrm{rev}}$, $P_{\mathrm{rev}\_\mathrm{end}}$ into the RTB packet.14 **else if** be on the road between S and $S_{\mathrm{pre}}$.15  Set the mode flag of RTB packet to 2 (means start the reverse selection).16  Choose the next corner as the optimal position $P_{\mathrm{opt}\_\mathrm{rev}}$ in the reverse direction17  Update $P_{\mathrm{opt}\_\mathrm{rev}}$ in the RTB packet.18 **Else**19  Set the mode flag of RTB packet to 1 (means start the normal selection).20  Determine the optimal position $P_{\mathrm{opt}\_\mathrm{norm}}$ in the message propagation direction according to **Definition 1.**21  Update $P_{\mathrm{opt}\_\mathrm{norm}}$ in the RTB packet.22 Broadcast the RTB packet.23**Phase 3. Relay-Node Selection Phase:**24 **if** the mode flag of RTB packet is 325  Start EPBP with $P_{\mathrm{opt}\_\mathrm{norm}}$ as the optimal position, $n_{\mathrm{norm}}\in N$ is not blocked by Ο is selected as the relay-node in the message propagation direction.26  Simultaneously, start EPBP with $P_{\mathrm{opt}\_\mathrm{rev}}$ as the optimal position, and $n_{\mathrm{rever}}\in N$ is not blocked by Ο is selected as the relay-node in the reverse direction.27 **else if** the mode flag of RTB packet is 228  Start EPBP with $P_{\mathrm{opt}\_\mathrm{rev}}$ as the optimal position.29  $n_{\mathrm{rever}}\in N$ is not blocked by Ο is selected as the relay-node.30 **Else**31  Start EPBP with $P_{\mathrm{opt}\_\mathrm{norm}}$ as the optimal position.32  $n_{\mathrm{norm}}\in N$ is not blocked by Ο is selected as the relay-node.33**Relay-node selection finished**

Next, we take Nodes S_3_ and S_4_ in Figure 1 as an example to illustrate our proposed method, and assume that Node S_3_ is used as a sender to start message broadcasting. According to Definition 1, Node S_3_ determines Point E as the $P_{\mathrm{opt}\_\mathrm{norm}}$. Then, we made a circle with Point E as the center and the distance between Node S_3_ and Point E as the radius, EPBP was performed on the circle as shown in Figure 1, and Node S_4_ was selected as the relay-node. After Node S_4_ receives the message from Node S_3_, as a new sender it determines that road section ① and ② are both vacant according to Definition 2. Then, it starts both the normal selection and the reverse selection: 

The initial sender S_4_ of the reverse selection chooses Point A as the terminal point of the reverse selection, chooses the corner (Point B) as the optimal position for the reverse selection, and selects Point G as the optimal position for the normal selection according to Definition 1. Then an RTB packet was broadcasted by Node S_4_ to inform nodes within its communication range that both the reverse selection and the normal selection were started at the same time. Finally, Node S_7_ was selected as the relay-node in reverse selection and Node S_8_ was selected as the relay-node in the normal selection. After that, Node S_7_ as a sender only performs the reverse selection, and Node S_8_ as a sender only performs the normal selection.

### 4.2. Criterion of Classifying Road Structures

In this subsection, we define a criterion to classify three typical road structures (junction, straight road, and curved road):

In previous works [25,26], the broadcasting in junction scenarios is completed through two-hop relay-node selection, and its message propagation direction is multidirectional. In curved road scenarios, there will be both normal and reverse relay-node selection for broadcasting, and the message propagation is bidirectional. To achieve full coverage of RoI, the priority of judgment for the criteria of road structures is junction, curved road, and straight road.

**Definition** **3.** *Junction scenario is a scenario in that there exists a junction in the communication range of the sender in the message propagation direction.*

It is widely accepted that the criterion for judging whether a road structure is a straight road or not is whether the line-of-sight condition exists. We define a curving rate to facilitate the definitions of the curved road and the straight road, as follows.

**Definition** **4.** *The curving rate β is expressed as*

(1) $$\beta =\frac{l}{R}$$

*where l is the length of the road within the communication range of the sender in the message propagation direction, and R is the communication radius.*

Based on the definition of curving rate, we give the definitions of curved road and straight road.

**Definition** **5.** *Curved road scenario is a scenario that $\beta >\beta_{\varepsilon }$ when no junction exists in the communication range of the sender in the message propagation direction, where $\beta_{\varepsilon }$ is a threshold.*

**Definition** **6.** *Straight road scenario is a scenario that $\beta \leq \beta_{\varepsilon }$ when no junction in the communication range of the sender in the message propagation direction.*

We discuss the value of threshold $\beta_{\varepsilon }$ based on whether obstacles on the roadside affect the line-of-sight propagation. When obstacles on the roadside affect the line-of-sight propagation of the message, the road will have at least one roundabout. For this circumstance, the road length must be more than twice the road width w beyond the communication radius, that is,

(2) $$\beta_{\varepsilon }=\frac{l_{\varepsilon }}{R}$$

where $\left(l_{\varepsilon }>R+2*w\right)$.

### 4.3. Adaptive Relay-Node Selection Method

In this subsection, we design an adaptive relay-node selection method based on the criterion of road structures, combining the relay-node selection method in curved road scenarios with obstacles proposed in Section 4.1 and an improved EPBP-based junction relay-node selection method in this subsection.

The termination condition of message broadcasting is to achieve complete coverage of RoI. That is, all ends of the roads at the RoI boundary are covered by the broadcasting message. Moreover, in order to avoid the multiple coverage of a message on one road section, the termination condition in junction scenarios is that the message covers the RoI boundary, or that the branch has been covered by the same message.

The EPBP-based junction relay-node selection method [26] includes a junction phase and a branch phase. It is suitable for urban scenarios where each branch of junctions is a straight road. Two types of nodes are selected successively as relay-nodes in the junction phase and the branch phase, which are closest to the center point of the junction and to the farthest point in the branches. However, in the real world, the branch of the junction, e.g., Junction J_1_ in Figure 1, may not be a straight road.

Therefore, we improve the EPBP-based junction relay-node selection method as follows. In the branch phase, first, the sender of the branch phase, i.e., the relay-node at the center of the junction, uses GIS information and the criterion of road structures to determine the road structure of each branch when it enters the branch phase. Then, according to the judgment result, a method suitable for the structure of branch is selected to complete the relay-node selection in the branch phase.

**Figure 2:**
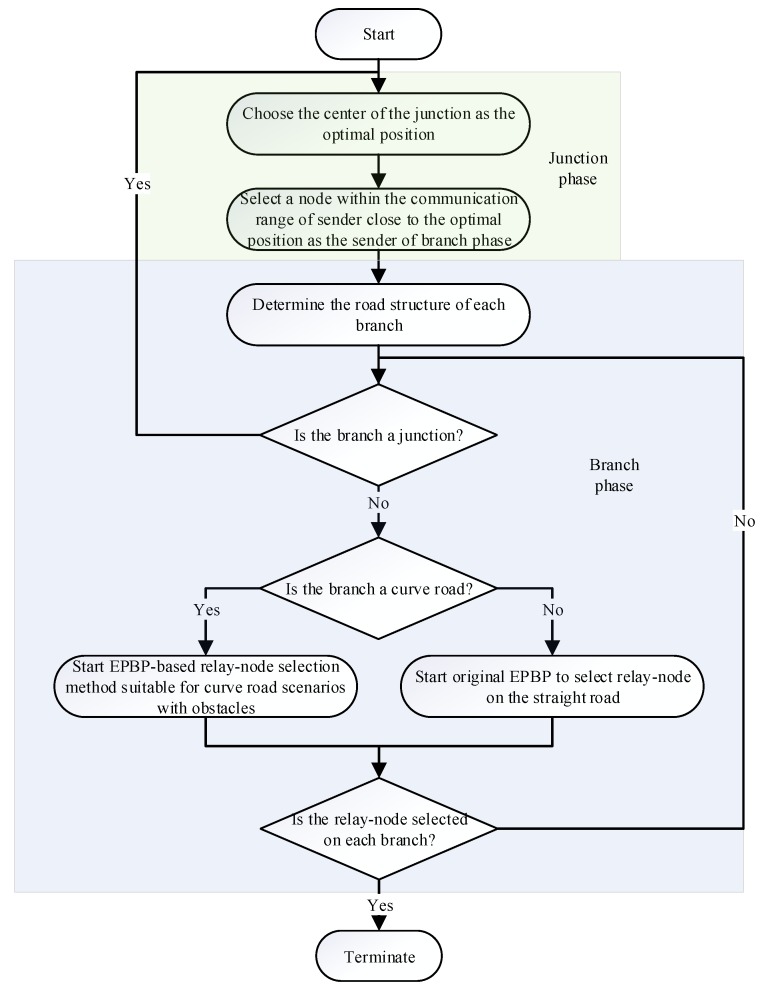
Flow diagram of the improved exponent-based partitioning broadcast protocol (EPBP)-based junction relay-node selection.

The flow diagram of the improved method is shown in Figure 2. The improved method realizes the adaptive relay-node selection in the branch phase. Compared with the original method [26], it has stronger robustness in real-world scenarios.

**Figure 3:**
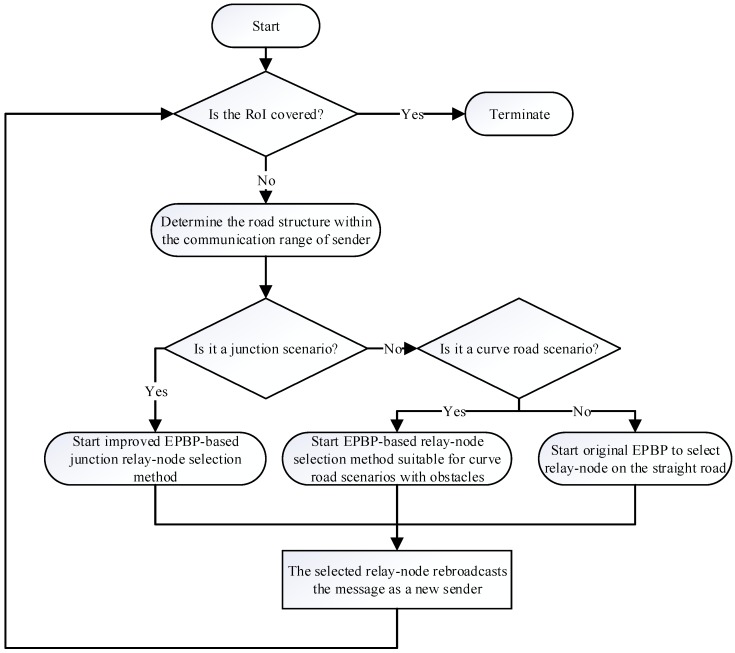
Flow diagram of adaptive relay-node selection (ARNS).

The adaptive relay-node selection mechanism is shown in the flowchart in Figure 3. First, ARNS determine whether the broadcast completely covers RoI. If a full coverage of RoI is not achieved, the criterion of road structures is adopted to judge the road structure within the current communication scenario. If the road structure is judged as a junction scenario, we adopt the improved EPBP-based junction relay-node selection method to realize relay-node selection in the current scenario; if the judgment result is a curved road scenario, we use the method proposed in Section 4.1 to select a relay-node in the current scenario; if the judgment result is a straight road scenario, we directly adopt the intersection of the sender’s communication boundary and road in the message propagation direction as the optimal position to implement straight road relay-node selection through EPBP. What is more, to ensure the security of message transmission, a caching optimization method [41] is used for each vehicle.

## 5. Results and Analysis

To prove the effectiveness, simulations were conducted on a real-world map shown in Figure 1, which is a part of the urban map in Zhangjiajie city, Hunan Province, China. In addition, to reflect the real-time advantages with the black-burst, ARNS was compared with a beacon-based method that uses RTAD [20] to select relay-nodes in urban scenarios and the GPSR method [19] on the curved road combined with the adaptive mechanism proposed in this paper. Additionally, a black-burst-based method was also used for comparison, which substitutes EPBP with 3P3B in ARNS (called the 3P3B-based method), to verify ARNS’ improvement. These results and analysis are presented in Section 5.2.

In addition, to demonstrate the advantages of considering obstacles in curved road scenarios, we compared ARNS with the complete relay-node selection method [25], which is an EPBP-based relay-node selection method well-qualified in the curved road scenarios without considering obstacles. These results will be discussed in Section 5.3.

### 5.1. Introduction of Evaluation

We simulated these above approaches in VANET using MATLAB with the Monte Carlo method [42]. Since we focused on the relay selection in the link level, the simulation environment just includes the 802.11p MAC layers. The major simulation parameters of VANET are given in Table 1, and are identical to those used in [20,23,25].

In each simulation, Node S_0_ was used as the original sender. The intersections of each road and RoI boundary were used as the terminal points of broadcast on this road. Since the roads in Figure 1 have different widths, for ease of expression, we classified them with the number of the lanes $n_{\mathrm{lane}}$ in both directions ($n_{\mathrm{lane}}$= 2,4,6), and vehicle density λ in this paper is defined as the vehicle density on a single lane.

**Figure 4:**
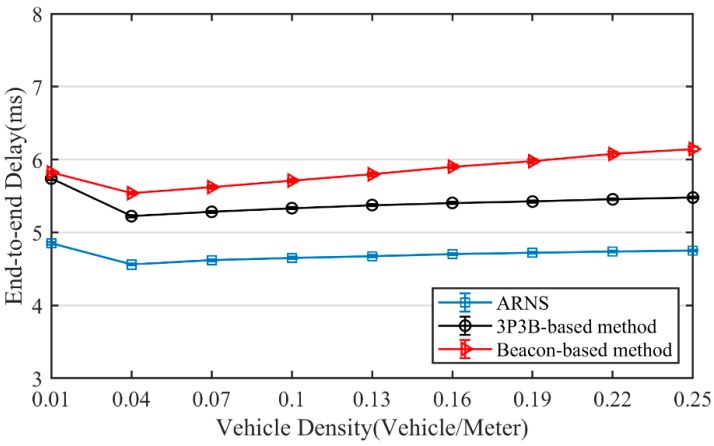
End-to-end delay.

In order to assess the performance of ARNS under a wide range of vehicle densities, we set the minimum interval between vehicles to be 4 m, and the minimum number of vehicles within communication range to be two vehicles. Thus, when the communication range was set to 200 meters, the lowest vehicle density was 0.01 vehicles/meter and the highest was 0.25 vehicles/meter. The vehicles were located randomly following the Poisson distribution with $\lambda n_{\mathrm{lane}}$. The maximum speed $v_{\max}$ of vehicles complies with the rule related to safe inter-vehicle distance [43,44]. Note that the inter-vehicle distance is defined as the distance between the heads of the adjacent vehicles. Each vehicle chose a random speed following a uniform distribution in $\left[\frac{1}{2}v_{\max},v_{\max}\right]$ at the beginning of the simulation, and kept the chosen speed during the simulation. Lane change and overtaking were not modeled for vehicle movement. From the simulation results shown in Figure 4, a single simulation duration, i.e., the end–end delay, is less than 6.2 ms, and the maximum movement distance of a node is 0.21 m corresponding the vehicle speed of 120 km/s. Thus, the above assumptions about the vehicle running are reasonable. The experimental environment was simulated in MATLAB, the same as [25], because the conclusion in [45] pointed out that the vehicle movement has little influence on the relay-node selection.

End-to-end delay and packet delivery ratio (PDR) are metrics widely used to evaluate the efficiency and reliability of message broadcasting in IoV [21,22,23,24,25,26,27]. In addition, a metric called maximum hops was proposed to evaluate the reliability of end-to-end delay. The metrics of broadcast coverage, partition delay, and contention delay were used to measure the improvement of considering obstacles. In this section, we show the comparisons of all method schemes in terms of six metrics: end-to-end delay, partition delay, contention delay, PDR, maximum hops, and broadcast coverage. The definitions of the metrics are described below.

**End-to-end delay**$T_{\mathrm{end}}$ is expressed as a total delay from the instant when Node S_0_ starts broadcasting to the instant when RoI is completely covered. $T_{\mathrm{end}}$ is the sum of one-hop delay. In the black-burst-based methods, the partition delay $T_{\mathrm{part}}$ and the contention delay $T_{\mathrm{cont}}$ dominate the one-hop delay. Thus, in the results of Section 5.3, $T_{\mathrm{part}}$ and $T_{\mathrm{cont}}$ are used to demonstrate the improvement of ARNS in the curved road scenarios. $T_{\mathrm{part}}$ is expressed as an average value of the partition delay in each hop, and $T_{\mathrm{cont}}$ is expressed in the same way.

**PDR** is expressed as a ratio of the number of successful broadcasting messages to the total number of simulations. Successful broadcasting means that no packet loss occurs during the entire broadcasting process.

**Maximum hops**$N_{\mathrm{maxhops}}$ is expressed as the maximum number of hops that a message is broadcasted from Node S_0_ to the terminations of RoI.

**Broadcast coverage**$\gamma_{\mathrm{cov}}$ is expressed as a ratio of the length of the road covered by broadcasting to the length of the entire road.

### 5.2. Evaluations of ARNS

In this subsection, we compare ARNS with RTAD and the 3P3B-based method in the same environment. We show the advantages of ARNS in three aspects, including end-to-end delay, maximum hops, and PDR. The simulation results show as follows.

Figure 4 shows the end-to-end delay obtained by each method with varying vehicle density. RTAD has the largest delay as it needs more hops to complete message broadcasting. In contrast, ARNS has the lowest delay as it costs the fewest hops by adaptively selecting the relay-nodes. Furthermore, we can see that, as vehicle density increases, end-to-end delay first decreases and then increases. The decrease is because message broadcasting can be implemented with fewer hops when vehicle density gets higher. The increase is due to the larger contention delay because of more nodes in the contention process.

**Figure 5:**
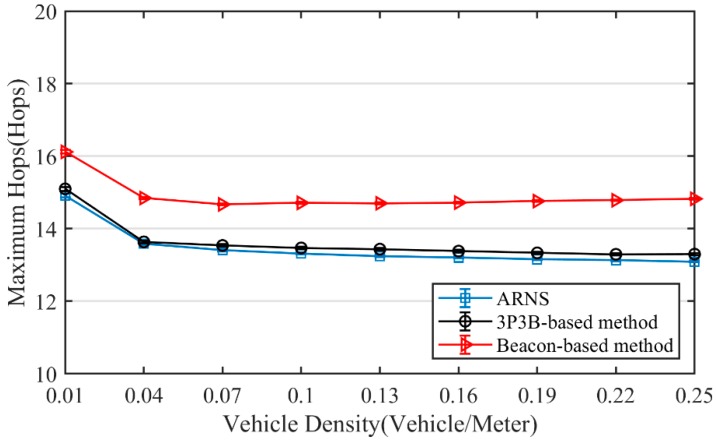
Maximum hops.

In Figure 5, the maximum hops of three methods are depicted to indicate the reliability of end-to-end delay shown in Figure 4. RTAD has the most hops as it selects corners as the optimal positions in curved road scenarios. In contrast, ARNS has the least hops since it improves the location of the optimal position. Moreover, with the increase of the vehicle density, the maximum hops of ARNS declines in a stable trend, while the maximum hops of the beacon-based method are already saturated. 

**Figure 6:**
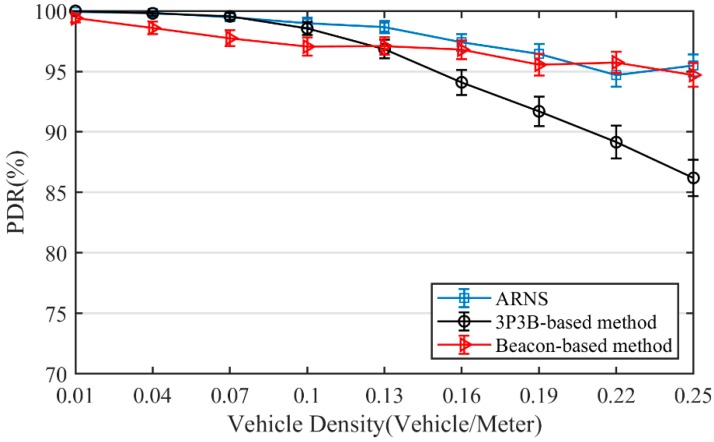
Packet delivery ratio (PDR).

Figure 6 presents the PDR of the three methods. It can be clearly seen that, as vehicle density ascends, PDR declines. PDR of ARNS is better than that of both the 3P3B-based method and the beacon-based method. Additionally, PDR of ARNS is more stable than the other two. The reasons can be derived as follows. Firstly, for the beacon-based method, nodes in its routing table may travel out of the communication range during the beacon interval, resulting in the loss of message packets. In this case, we will re-transmit. However, if the number of re-transmissions reaches the maximum times, the message packet is still missing, then the broadcast is considered as a failure. However, the relay-node selection of ARNS is real-time, so ARNS is more stable than the beacon-based method. Secondly, compared with the 3P3B-based method, the partition phase of ARNS selects a smaller segment than the based-3P3B method. Then fewer nodes participate in the random contention phase. This results in the gain for PDR of ARNS. Therefore, PDR of ARNS is the most stable among the three methods.

### 5.3. Evaluations of ARNS in the Scenario with Obstacles

In this subsection, we simulated ARNS and the complete relay-node selection method on the curved road [25], which do not consider obstacles, to show the advantages of considering obstacles in three aspects of broadcast coverage, partition delay, and contention delay. The simulation results of partition delay and contention delay indicate that the proposed method ARNS significantly reduces the delay of the relay-node selection.

**Figure 7:**
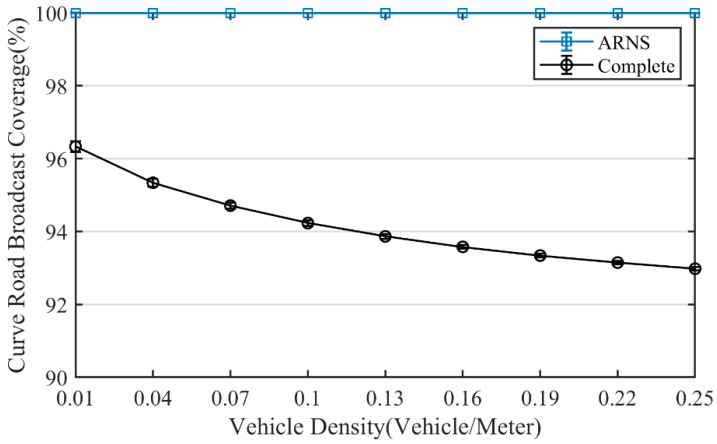
Broadcast coverage.

As shown in Figure 7, the broadcast coverage of the curved road method decreases with the increase of vehicle density. It was because when vehicle density was low, the curved road method selects relay-nodes along the curved road to achieve broadcast coverage. When vehicle density increases, the curved road method gradually reduces the number of times to select relay-nodes along the curved road. However, the number of times to select relay-nodes across the curved road increases gradually (in Figure 1, for example, Node S_3_ selects Node S_4_ as a relay-node). Thus, the broadcast coverage of the curved road method gradually decreases. 

**Figure 8:**
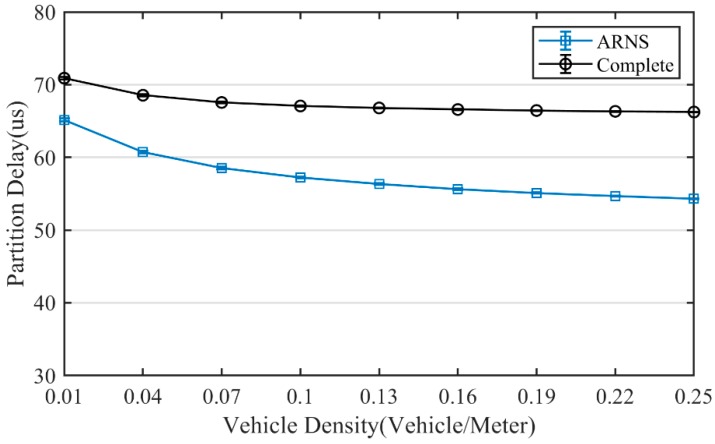
Partition delay.

**Figure 9:**
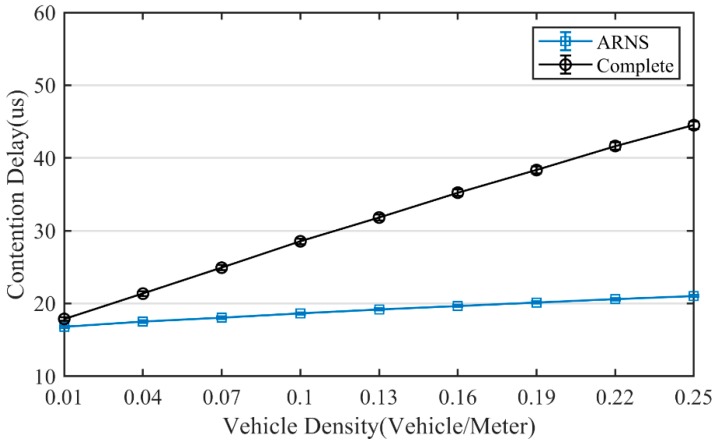
Contention delay.

**Figure 10:**
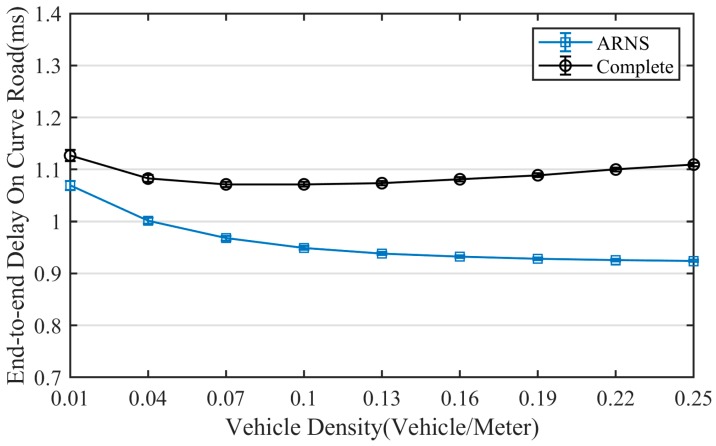
End-to-end delay on the curved road.

Choosing different optimal positions in the same scenario will cause different partition delays and contention delays. Thus, as shown in Figure 8 and Figure 9, ARNS has obvious advantages in the partition delay and contention delay. As vehicle density increases, these advantages become more apparent. At a high density of 0.25 vehicle/meter, the partition delay of ARNS was reduced by 16.4% compared with the complete method, while the contention delay of ARNS was reduced by 52.2%. These results are reflected in the end-to-end delay as shown in Figure 10, and compared with the complete method, ARNS can reduce end-to-end delay on a curved road by up to 16.3%.

## 6. Conclusions

In this paper, we proposed the ARNS method for the relay-node selection in complex road scenarios. To the best of our knowledge, it is the first time developing an adaptive relay-node selection mechanism considering the road structure within the communication range of the sender in each hop. ARNS adopts the favorable relay-node selection method according to the road structure. In addition, the effect of obstacles was considered. It was demonstrated through simulation that ARNS is superior to methods based on 3P3B [23] and RTAD [20] in terms of the end-to-end delay and PDR, and superior to the complete method [25] in terms of the broadcast coverage and one-hop delay. In a real-world road scenario, we showed that ARNS reduces end-to-end delay by at least 13.8% compared to the beacon-based method, and the broadcast coverage of ARNS was increased by 3.6–7% compared with the complete method.

In the future, we plan to extend our work to relay-node selection on 3D road structures, such as overpass structures and parking lot structures, and utilize AI [46,47,48,49,50,51] to optimize the method [52,53] in complex 3D scenarios.

## Figures and Tables

**Table 1:** Major communication parameters.

Parameters	Default Values
Communication Range	200 m
DIFS	58 μs
SIFS	32 μs
Slot Time	13 μs
Transceiver’s Switching Time	1 μs
Beacon Interval	100 ms
Bit Rate	18 Mbps
RTB Packet Size	20 Bytes
CTB Packet Size	14 Bytes
ACK Size	14 Bytes
Message Packet Size	500 Bytes
Confidence Interval	95%
Simulation Times	2000
